# Synthesis, characterization of hybrid nanocomposite material and fabrication of their advance membrane for removal of antibiotics from water

**DOI:** 10.1371/journal.pone.0332699

**Published:** 2025-09-18

**Authors:** Fazal Suhrab Gul, Hayat Ullah, Ali Umar, Maliha Sarfraz, Muhammad Salman Khan, Misbah Ullah Khan, Muhammad Saleem Khan, Shifa Shafique, Lala Gurbanova, Abdul Wadood, Hanan A. Ogaly, Fatimah A. M. Al-Zahrani

**Affiliations:** 1 Department of Chemistry, Hazara University, Mansehra, Khyber Pakhtunkhwa, Pakistan; 2 Department of Chemistry, University of Okara, Okara, Pakistan; 3 Department of Zoology, Faculty of Life Sciences, University of Okara, Okara, Pakistan; 4 Department of Zoology, Wildlife and Fisheries, University of Agriculture Faisalabad, Sub Campus Toba Tek Singh, Pakistan; 5 Center for Nanosciences, University of Okara, Okara, Pakistan; 6 Department of Applied Biosciences, Kyungpook National University, Daegu, Republic of Korea; 7 Department of Life Sciences, Western Caspian University, Baku, Azerbaijan; 8 Department of Biochemistry, Abdul Wali Khan University Mardan, Pakistan; 9 Chemistry Department, College of Science, King Khalid University, Abha, Saudi Arabia; Wadi International University, SYRIAN ARAB REPUBLIC

## Abstract

In this work, a selective membrane for antibiotics removal from water was fabricated. The ingredients utilized for membrane fabrication include functionalized TiO_2_ nanoparticles (NPs), poly methyl methacrylate (PMMA) and poly vinyl imidazole (PVIM). Firstly, TiO_2_ nanostructure’s surface was functionalized through APTES. The layer of functional monomers methyl methacrylate (MMA) and VIM (vinyl imidazole) was grafted onto TiO_2_ surface through controlled radical polymerization technique. The grafted material was fabricated into membrane with embedded selectivity utilizing molecular imprinting method. The material was diagnostically analyzed with Fourier Transform Infrared-Attenuated total reflection (FTIR-ATR) spectroscopy and NMR (Nuclear Magnetic Resonance) technique, which confirm functionalization of nanomaterial surface. The grafted layer around the nanoparticles was visualized with FESEM. The hybrid membrane shows excellent and promising results against removal of antibiotics from aqueous medium. The fabricated membranes via Molecular imprinted polymer (MIP) technique were applied for the selective removal of antibiotics (ciprofloxacin) from real water samples. The removal efficiency and selectivity of the hybrid membrane was monitored via UV visible spectroscopy.

## 1. Introduction

The bioavailability of drugs in the human body is only 70–80% while 20–30% excreted from the body in its parent form [1,2]. This un-metabolized (parent) fraction of the medicines as well as unregulated affluents discharge from the pharmaceutical industries exacerbates the danger posed to the environment ultimately finds its way into the freshwater streams through the mechanism of leaching particularly in the third world countries. In fact, the effectiveness of medicine as curing agent is linked to the its biologically available quantity [3]. All these activities inject drugs in very miniscule quantities into the bodies of human and microbes also, which augmented adaptation of the microorganism with the drugs and generate resistance among the population. Such resistance in case of antibiotics called antibiotics resistance (AR) [4,5]. Most of the materials reported in the literature, no doubt performs its intended purpose but selectivity is an issue and also non-regenerative. Therefore, much attention needs to be focused on the development of selective and regenerative materials [6]. In ancient times activated carbon was widely used as water purification material but there were some drawbacks and limitations like high production/regeneration cost and non-specificity. Many other physical purification methods are also in practice like coagulation/sedimentation, flotation [7], and membranes [8–10] etc., but each has its own merits and demerits [11]. Mohan *et al*, successfully used iron-titania composite material for the degradation of the norfloxacin (NF). Their claim of 100% degradation may be prone to intellectual rationale [12]. The nanocomposite shows activity for degrading NF, but it may not be able to interact with other antibiotics due to the attenuated band gap of the composite after doping of TiO_2_. The composite material suffers from shortcomings of regeneration and cost of the process. The processability of this material may not find use in practical application due to the hazardous sludge generation after application [13,14]. R Molinari et al, tried in this direction and degraded the congo red dye through membrane loaded TiO_2_-P25 photocatalyst [15]. The author noted that rate of degradation of the dye was lowered due to the adsorption of the dye on the catalyst surface as well as temperature variation. Derakhsheshpoor *et al*. evaluated the separation of amoxicillin from pharmaceutical wastewater using a polysulfone nanofiltration membrane [16]. Javier Benitez et al., evaluated the real time application of ultrafiltration and nanofiltration membranes for removal of amoxicillin, naproxen, metoprolol, and phenacetin from wastewater [17]. On the contrary, despite membrane versatility and application’s plethora, the technique still suffers from fouling, selectivity, and regeneration problems [18–23]. Numerous researchers have attempted to counter problems in membrane applications by making the surface hydrophilic, but with little benefits. Therefore, we believe that combining hydrophilic character with the imprinting strategy may solve these problems. The hydrophilic character due to the charge nature of metal oxide surface and molecularly imprinting strategy for selectivity may prove beneficial in this regard [24–26]. The mechanical strength of the material originated with the good adhesion of the reinforcement to the matrix, which demands enough distribution of the reinforcement in the matrix. The “grafting from” method facilitates efficient dispersion yielding mechanically strong material. In the grafting form, approach particles are dispersed in the solvent and matrix is netted from the surface of particles countering aggregation. Another important factor of mechanically strong polymeric membrane is control over molecular weight of the polymer for which controlled radical polymerization is mostly applied.

The aim of the work is to synthesize hybrid composite materials with selective and regenerative capabilities. Fabricated their membranes for the removal of antibiotics from water bodies.

## 2. Experimental

### 2.1. Material

Functionalized TiO2 nanoparticles, methyl methacrylate (MMA) (Uni chem), Vinyl imidazole (VIM), triethylamine (99%) (TEA), N, N, N, N, N, pentamethyl diethylenetriamine (PMDETA), α-Bromoisobutyryl bromide (Bi-BB), Copper (I) bromide (Cu(I)Br) (99.999%), copper II bromide (Cu(II)Br) (99.99%), tetrahydrofuran (THF) dimethyl formamide (DMF) and methanol were purchased from Sigma Aldrich. Monomer was purified bypassing through an alumina column to remove inhibitor. All solvents of analytical grade were used.

### 2.2. Immobilization of Si-ATRP initiator on TiO_2_ surface (Br@APTES@TiO_2_) (A, B, C)

2.0 mmol of pre synthesized functionalized TiO_2_ NPs [27] and 15 mL of THF was taken in a 2-neck reaction flask and dispersed through sonication. Triethylamine (7.5 mmol) was added to the flask, and the reaction mixture was stirred at 0°C (in an ice bath) for 60 minutes. During stirring at 0^o^C different amounts of the initiator (Bi-BB) i.e., 4.5 mmol, 5.37 mmol, 6.24 mmol were injected dropwise into the reactor through a syringe and allowed the contents to react further for 2 hours at 0°C. Furthermore, the contents of the reaction flask were stirred for an additional 10 hours at 25°C. The reaction was stopped, and the final product was washed with THF, methanol and water in succession to remove unreacted material.

### 2.3. Grafting of PMMA-co-VIM polymer brushes onto TiO_2_ NPs via surface-initiated atom transfer-radical-polymerization (Si-ATRP)

In an experiment the ingredients including Cu(I)Br: PMDETA: MMA in the molar ratio of 1:10:1000 was taken in the reaction vessel along with 15 mL of DMF. The contents of the reactor were swirled for 1 hour. After that the specific molar solution of Cu (II)Br: VIM)) in different molar ratio of 1:100, 1:200, 1:300, and 1:400 in 5 ml DMF was injected into the reactor. Then, a suitable amount (0.453 mmol) of macro-initiator labelled as A, B, and C in Table 1 dispersed in 5 ml of DMF were transferred to the reactor. The reactor was sealed with rubber septum and purged by N_2_ gas for 30 min. The reactor was allowed for 60 min at room temperature to swirl and then shifted to the pre heated oil bath (75 °C) and reacted for 18h. The reaction was stopped with the appearance of the turbidity by exposing the contents to air for conversion of Cu(I) into Cu(II). The contents were precipitated into enough diethyl ether. The precipitate was washed with methanol followed by acetone to remove the unreacted reagents and pre-polymer. The material was dried overnight under vacuum. Different samples were prepared according to Table 2.

**Table 1:** Feed molar ratio of macroinitiator (Br@APTES@TiO_2_).

Sample Code	APTES@TiO_2_ mmol	Bi-BB mmol	TEA mmol
A	2.0	4.5	7.5
B	2.0	5.37	7.5
C	2.0	6.24	7.5

**Table 2:** The feed ratio of hybrid nanocomposite.

Sample code	Macroinitiator mmol	Cu(I)Br mmol	Cu(II)Br mmol	Ligand mmol	MMA mmol	VIM mmol
D	0.453	0.0419	0.0419	0.419	41.9	4.19
E	0.453	0.0419	0.0419	0.419	41.9	8.38
F	0.453	0.0419	0.0419	0.419	41.9	12.57
G	0.453	0.0419	0.0419	0.419	41.9	16.76

### 2.4. Synthesis of hybrid nanocomposite’s membrane

0.5g of each sample D to G were taken in the beaker in DMF to solubilize the contents, yielding yellowish homogenous solution. The contents were stirred for 1 hour at 60 ^0^C. After 1 hr., it was poured into a clean petri dish. The petri dish was de-aerated in vacuum oven at 60 °C for 12 hours. Upon transformation into a semi-solid layer the petri dish was immersed in a coagulation bath (water) and through phase inversion process it transforms into a membrane and dries at room temperature. One of the samples labelled as G, was dispersed in the solvent along with the 5 mg of the template molecule (Ciprofloxacin) to create imprints in the resulting membrane. The template was removed from membrane using slightly acidic distilled water followed by methanol. The removal of template was confirmed by UV-visible spectroscopy (Table 3).

**Table 3:** Hybrid nanocomposite membrane.

Sample code	Nanocomposite	Templet (cipro)
M1	D (0.5g)	–
M2	E (0.5g)	–
M3	F (0.5g)	–
M4	G (0.5g)	–
M5	G (0.5g)	5 mg

The real time application was performed on H-Cell apparatus. (See supplementary information S1 and S2 Figs).

## 3. Results and discussion

### 3.1. FT-IR analysis

All samples were characterized by using parkin Elmer FTIR-ATR spectrometer. First the spectrometer was calibrated with 32 scans and resolutions were set at 4 cm^-1^ to produce the resulting spectra of the sample. All results were auto baseline in the transmittance mode before analysis.

**Fig 1:**
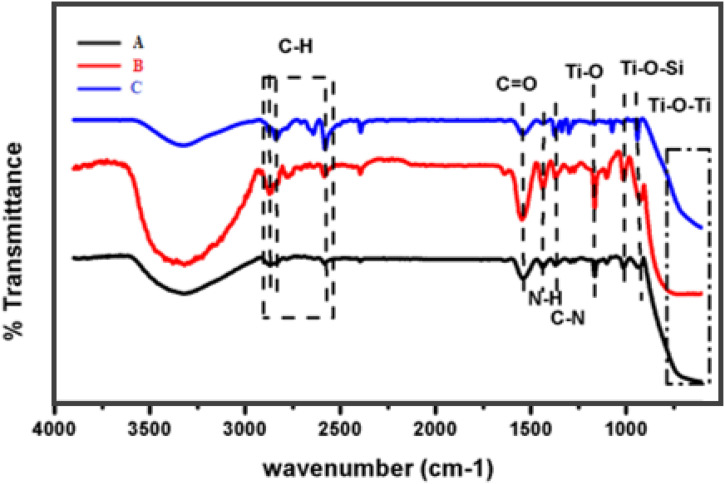
FT-IR spectra of sample A, B, and C.

The IR signal appears at 1680 cm^-1^, confirmed the presence of amide N-C = O bond formation because of a reaction between the NH_2_ group of covalently attached APTES and the bromine (Br) entity of the BiBB. The peak at 1535 cm^-1^, reveals N-H bond was also formed, confirming that reaction occurred completely shown in Fig 1. This vibration was influenced by the neighboring oxygen (O) of the carbonyl group due to hydrogen bonding and high electronegativity (E.N), as electronegativity affects the intensity of peak in the IR spectrum due to bond polarity. The N-H and C = O peaks of the macroinitiator were sharper and more intense when compared to APTES functionalized due to the presence of electronegative oxygen (O) and nitrogen (N). The mass of the attached atoms was an important factor in determining the IR frequencies. If the molar mass of the bonded substance is high, it will absorb lower IR frequencies. Exactly, when BiBB was linked with NH_2_, the N-H bond frequency shifts from 1569 cm^-1^ to 1535 cm^-1^. The peak at 1353 cm^-1^ was attributed to the geminal dimethyl group of the BiBB entity [28].

The peak at 3111 cm^-1^ was assigned to the H-N = C stretching vibration while the sharp peak at 3348 cm^-1^ was attributed to the vibration frequency of amine (N-H). However, the peaks at 1669 cm-1, 1493 cm^-1^, 1441 cm^-1^ were attributed to C = N, C = C and C-N stretching vibration of PVIM CH) group of PMMA was observed between 3000 cm^-1^ 2800 cm^-1^ [29–31].

**Fig 2:**
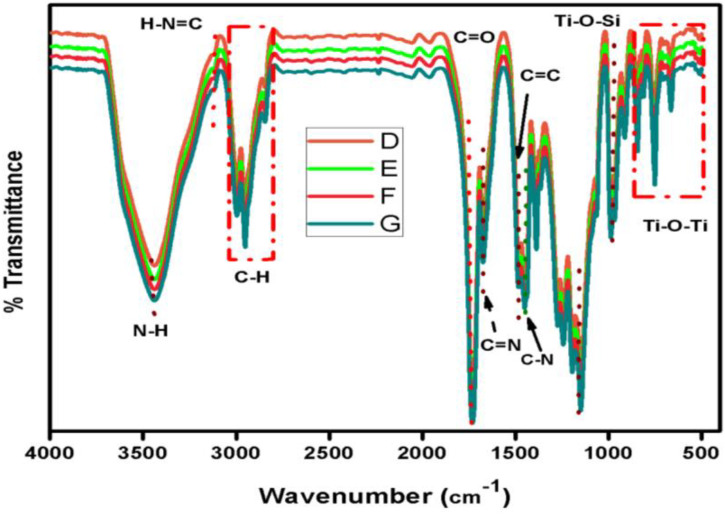
FT-IR spectra of sample D, E, F, and G of nanocomposite TiO2@PMMA@PVIM.

The stretching vibration at 1722 cm^-1^ and 1154 cm^-1^was attributed to carbonyl-group (C = O) and C-O-C in the PMMA molecule [32,33]. Fig 2 exhibited envelop in beneath 3000–2800 cm^-1^ spectral position range, attributed to the C-H stretching vibration band of –(CH_2_)n and CH_3_ species of PMMA. The stretching vibration at 3441 cm^-1^ is attributed to N-H vibration of NH_2_ group, mostly the vibration frequency of amine group and hydroxyl group usually appear at the same position. In FTIR, the analysis of the C = O band in the polymer mixture can provide valuable insights into the chemical changes occurring during chain propagation reactions. The observed relatively weak shift of the C = O band from 1730 cm^-1^ to 1722 cm^-1^ can be explained by the disappearance of the conjugation effect between the double bond of the corresponding atom (C = O) and the imidazole ring in the polymer mixture. The peak at 1272 cm^-1^and 1240 cm^-1^ were also attributed to C = N and C = C ring vibration of P(vinyl-imidazole) [34,35]. However, the asymmetric 1390 cm^-1^ symmetric vibration frequency of CH_2_ group has been observed. Another stretching vibration has been detected on 1184 cm^-1^ assigned to the Si-CH_2-_R bond vibration.

### 3.2. NMR analysis

NMR is an authentic and valuable technique used to determine the structure, dynamics, and other properties of molecules by studying the interaction of their nuclei with a strong magnetic field and radiofrequency radiation.

**Fig 3:**
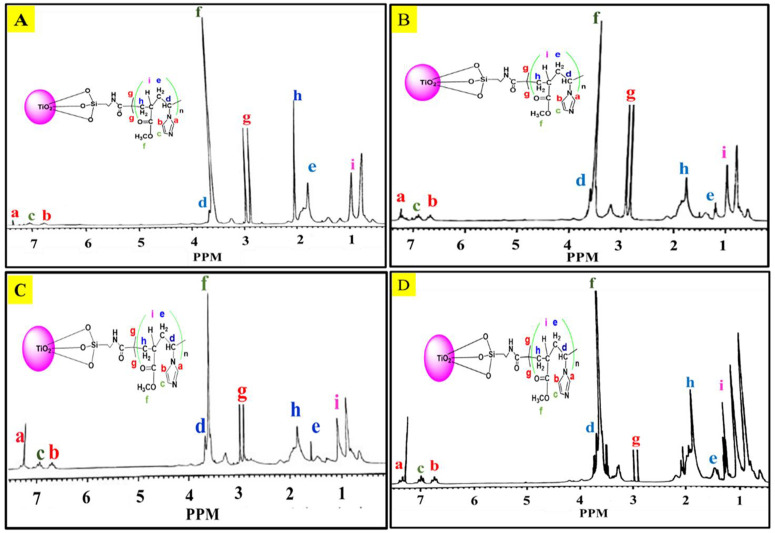
¹H NMR spectra of hybrid nanocomposites (Samples D–G) confirming PMMA–PVIM grafting on TiO₂.

Reference tetramethyl silane (TMS) as standard was selected based on its well-defined and easily identifiable resonances in the NMR spectrum. The reference standard was typically added in a small quantity (usually a few drops) to a separate NMR sample tube. The NMR solvent used for the reference should match the one used for the samples being analyzed and ensure that the NMR spectrometer is properly tuned and calibrated. This involves adjusting various parameters to optimize the signal-to-noise ratio and sensitivity of the NMR measurements. The NMR spectrum of the sample D, E, F, and G shown in Fig 3, illustrated signals at 2.9 ppm and 2.8 ppm corresponding to the methylene protons of the ethyl ester of BIBB moiety. While, at 3.75 ppm the methyl ester protons of the PMMA polymer, respectively. These chemical shifts help to identify and characterize the PMMA present in the sample, contributing to the understanding of its molecular structure. The signal that appears at 3.82 ppm was attributed to proton ^1^H of PVIM at potion (d). the signal of the proton of CH_3_ group of PMMA was detected at 1.2 ppm. The signal at 7.43 ppm, 6.92 and 6.87 ppm were assigned to the PVIM ring portion of the nanocomposite position at a, b, and c shown in Fig 3. The peak at 1.5 ppm (e) predicts the ^1^H of bridging carbon (C) of PVIM and PMMA [36]. According to the result of NMR analysis consider the copolymer of PPMA@PVIM successfully grafted on TiO_2_ NPs surface.

### 3.3. Morphological studies of TiO_2_ Nan-composite

For the surface morphological analysis of D, E, F, and G followed all the safety guidelines for operating the FESEM (**JSM-IT710HR Scanning Electron Microscope Made in Japan**). Calibrate the instrument, it was crucial for accurate measurements of features of the samples D, E, F, and G. Used the SEM’s stage controlled the position of sample correctly. It was essential to find a suitable area of interest before going ahead with detailed imaging. Configure the imaging settings for nanocomposite D, E, F, and G analysis.

**Fig 4:**
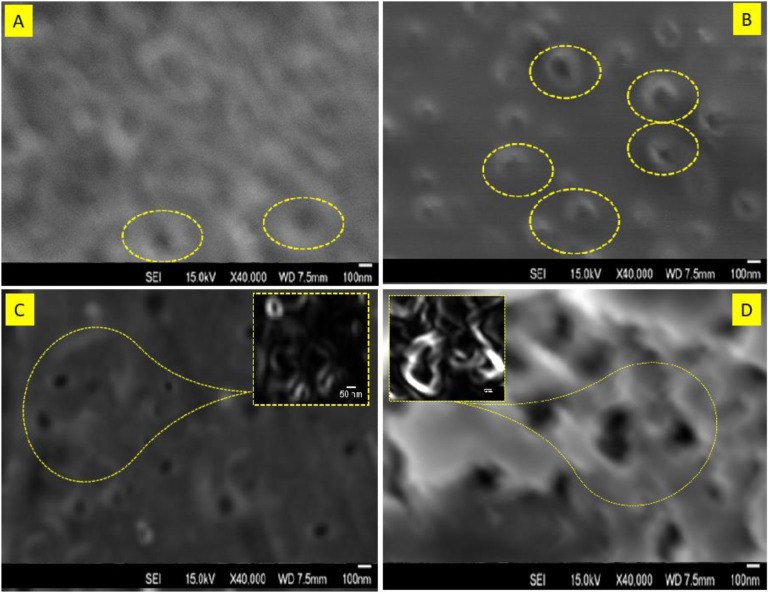
SEM images of TiO₂@PMMA@PVIM nanocomposites (Samples D–G) showing surface morphology.

Once the sample was aligned and the imaging settings were adjusted, started acquiring images of the sample. Then explored the surface morphology, of the sample was using various imaging modes and detectors (secondary electron, backscattered electron, etc.). It appears that Fig 4 shows the grafting of the polymers PVIM@PMMA on the surface of TiO_2_ nanocomposite. The SEM study investigated the effect of changing the feed ratio of Vinyl imidazole on the grafting density of the grafted polymers, Surprisingly, were not altered by changing the feed ratio of Vinyl imidazole. To visualize and clarify the polymer brushes, in this research work used a software called ImageJ. This software (ImageJ) is a widely utilized for image processing and analysis of different aspects, particularly in the field of scientific research. It allows us to analyze and quantify images, making it useful for tasks like measuring the size of polymer brushes. ImageJ helped to obtain quantitative data from the SEM images, which supported their observations and provided valuable insights into the polymerization process and nanocomposite structure [37].

### 3.4. UV-visible spectroscopy study for the detection of antibiotic

Prepare of standard solution of known ciprofloxacin concentrations 10-ppm. Set up a UV-visible spectrophotometer (**Analytik Jena Specord 200 Plus**) according to the manufacturer’s instructions. Use a cuvette and fill it with reference usually D.I water and the filtered water sample after passing through the hybrid nanocomposite membrane (if the concentration was expected to be within the detectable range). The operation was done in H-cell tool see (Supporting information).

Measure the absorbance of each solution at a proper wavelength within the UV-visible range, typically around 270–280 nm for ciprofloxacin (CP). Create a calibration curve by plotting the absorbance against the known concentrations of ciprofloxacin from the standard solutions. Measure the absorbance of the filtered water sample containing ciprofloxacin. In the case of M5 membrane it was a special membrane fabricated by MIP technique that was treated with two antibiotics to check the selectivity. After removing the template (CP) form membrane was confirmed by VU-Visible.

**Fig 5:**
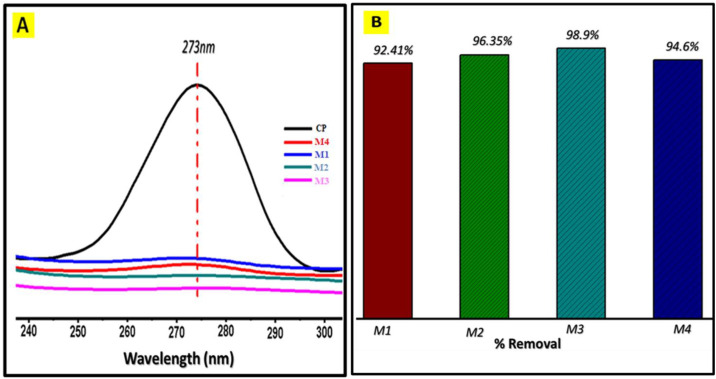
UV-Visible spectra of CP (A) and % removal of CP (B).

Fig 5 shows the % absorption of CP (A) and % removal (B) in different nanocomposite membranes labeled as M1, M2, M3, and M4.

**Fig 6:**
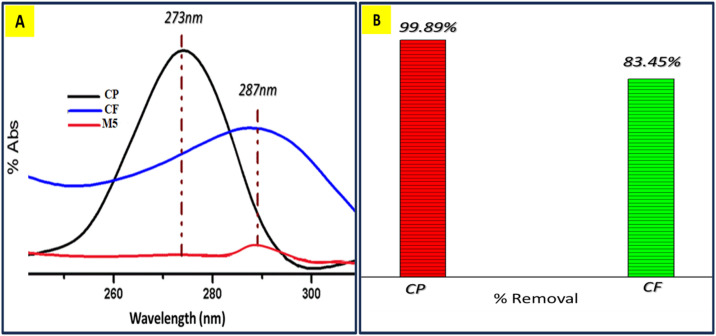
UV visible spectra of M5 (A) % Abs of antibiotics, (B) % Removal of antibiotics.

The nanocomposite membranes contain two polymers: hydrophilic PVIM (polyvinyl imidazole) and hydrophobic PMMA (polymethyl methacrylate). M1 membrane has less PVIM and more PMMA. As the molar ratio of PVIM increases from M1 to M3, the % removal of CP also increases. It means that with a higher proportion of PVIM compared to PMMA, the membrane’s ability to adsorb CP improves, because PVIM has excellent compatibility to CP. The presence of ring structure and NH functionality rather than PMMA. As the molar ratio of PVIM increases the % absorption of CP decreased from M1 to M3 and the percent (%) removal of CP increases (92.35%,96.4%, and 98.8%) respectively (Fig 6).

However, in M4, there was a higher fraction of the hydrophilic polymer PVIM and less PMMA but surprisingly, the % absorption of CP decreased and % removal mitigate in M4. The reason behind this unexpected result was the enhance rate of permeability, high water Flux and Applied pressure on membrane, could become unable to hold CP effectively during filtration depicted. In summary, the nanocomposite membrane’s adsorption behavior for CP was influenced by the molar ratio of hydrophilic PVIM and hydrophobic PMMA. An optimal balance between these two polymers was observed to be crucial for achieving the best adsorption performance. Fig 6 shows the molecular imprinted membrane (M5) using the same molecularly imprinted polymer (MIP) techniques exhibit significant improvement in the performance of the membrane compared to M4.

**Fig 7:**
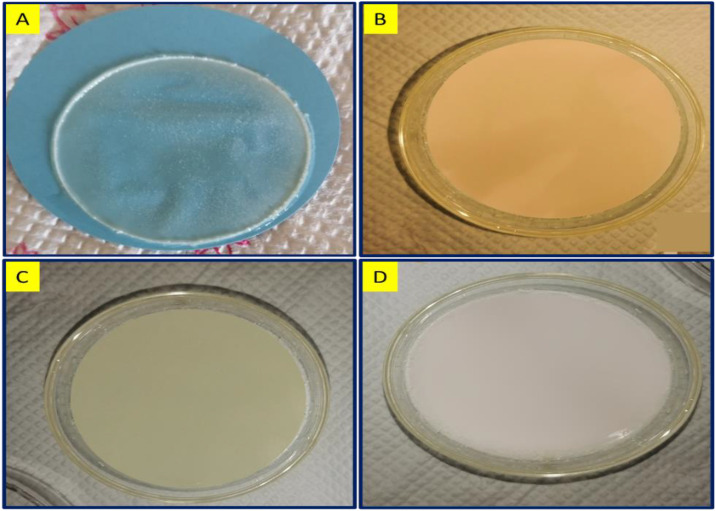
Visual view of nanocomposite membrane.


The molecular imprinted polymers are designed to have specific recognition sites of target molecules to mimicking natural receptors and leading to enhanced selectivity. Based on description, it seems that the MIP protocol introduced specific cavities (imprints) in the membrane that match the size and shape of the CP molecules. As a result, when the 10-ppm solution Cefixime (CF) and CP was passed through the membrane, CP not only interacts through physical forces as seen in M4 but was more effectively trapped inside the imprint cavities w.r.t CF. This induced fit mechanism ensures a much tighter and selective binding of CP molecules to the imprinted membrane. This improvement in selectivity and % removal of Cp was likely the reason behind the spectacular results observed with M5 compared to M4 shown in Fig 7.

$$Selectivity=\frac{KL,CP}{KL,CF}=\frac{1.047}{0.12}\approx 8.7$$ (1)

The CP imprinted membrane (M5) has the advantage of combining the physical adsorption present in M4 with the chemical recognition provided by the molecularly imprinted cavities, resulting in enhanced performance in selectively removing CP. Equation 1 show that M5 absorb CP 8.7 x more efficiently than CF. It’s worth noting that molecular imprinting techniques have been widely used to create specific recognition sites for various molecules, leading to improved selectivity and efficiency in separation and sensing applications. In this case, the unexpected but positive outcome. reinforces the benefits of using molecularly imprinted membranes for targeted removal of specific substances.

## 4. Conclusion

It concluded that the surface functionalization of TiO_2_ nanostructure was successfully modified by covalent of Si-ATRP initiator, (α-Bromoisobutyryl bromide Bi-BB). PMMA and PVIM polymers brushes onto functionalized TiO_2_ nanomaterial were grafted. The filtration for the removal of (antibiotics) was assessed by nanocomposite membranes (M1-M4) and also by smart/intelligent membrane(M5) under H-Cell tool. The results revealed that the functionalized nanocomposites and their membrane exhibited significantly improved water purification (removal of CP). The improved efficiency can be assigned to the presence of the grafted polymer chain not only facilitated the dispersion and stability of nanocomposite but also provide additional functional group for physical attachment to antibiotic. Therefore, the membranes exhibit spectatorial abilities, not only enhanced their % removal efficiency of CP as well as its selectively due to MIP techniques. These results highlight the effectiveness of combining hydrophilic PVIM, SI-ATRP grafting for dispersion/strength, and molecular imprinting for selectivity in creating advanced nanocomposite membranes for targeted antibiotic removal, offering potential for improved water purification.

## Supporting information

S1 Fig The Representative Image of H-Cell. (DOCX)

S2 Fig The H-Cell during real time operation. (DOCX)
