# Supplementary figures and images for: Synthesis, characterization of hybrid nanocomposite material and fabrication of their advance membrane for removal of antibiotics from water
Source: PLoS One. 2025 Sep 18;20(9):e0332699. doi: 10.1371/journal.pone.0332699 (PMC12445502; doi:10.1371/journal.pone.0332699)

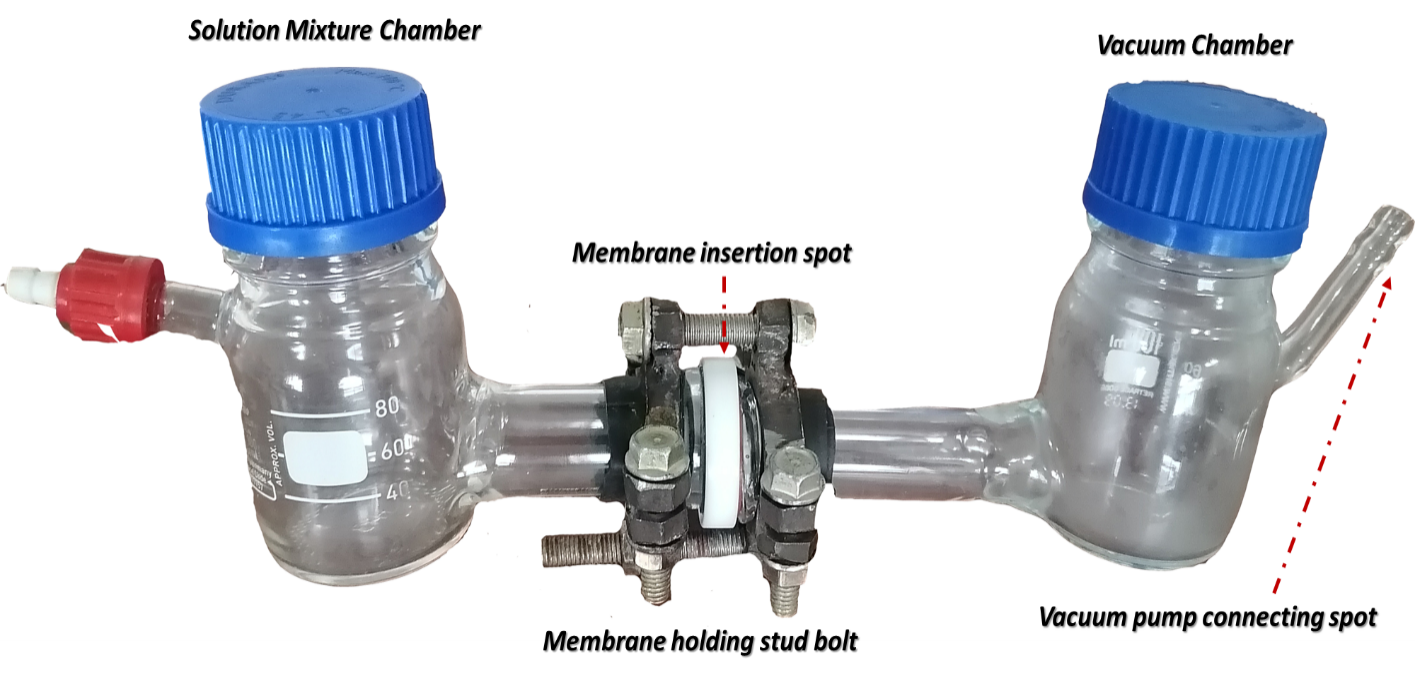


**Figure-S1:** The Representative Image of H-Cell

Supplement: S1 Fig — (DOCX) [file pone.0332699.s001.docx]

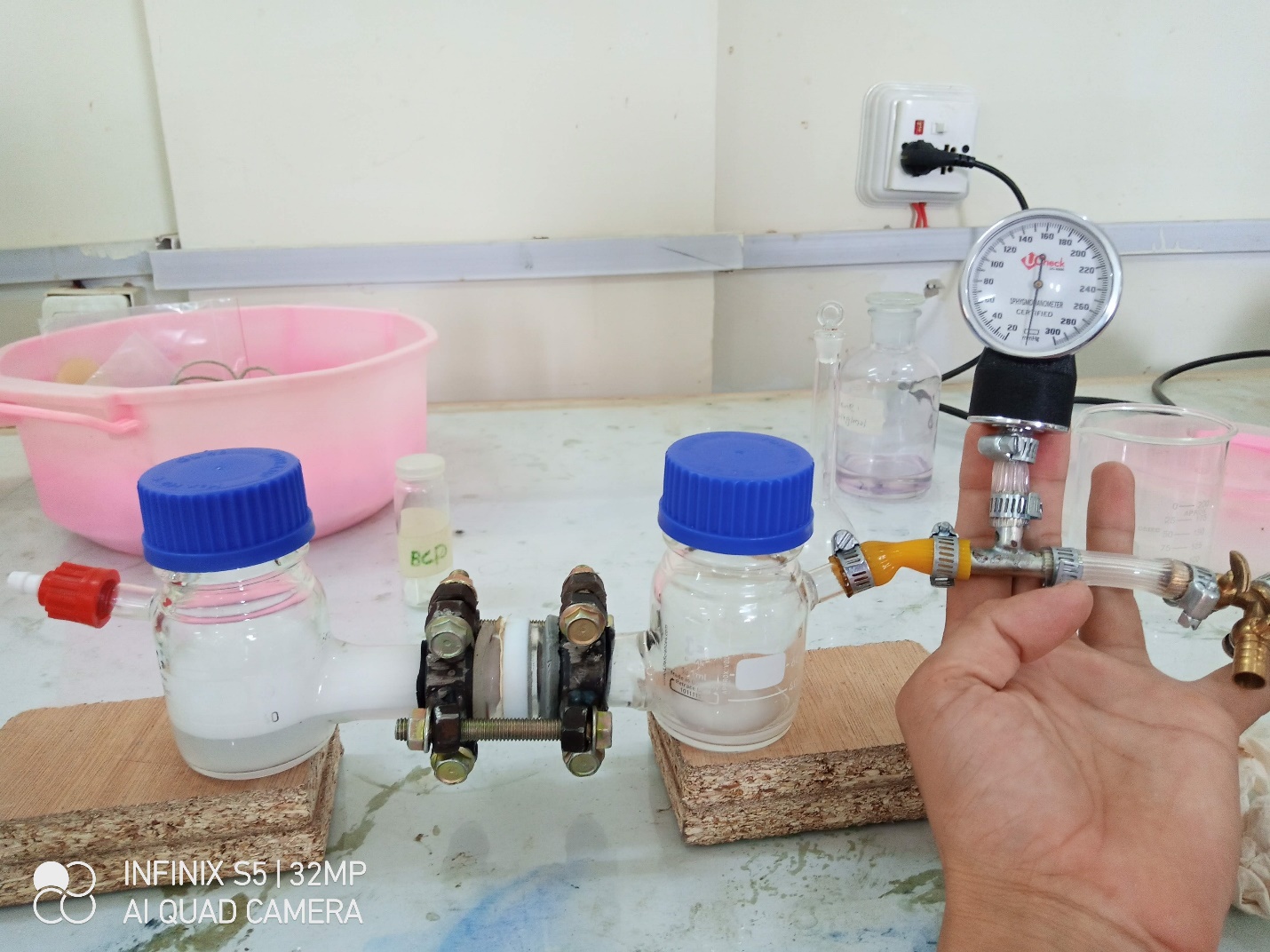


**Figure-S2:** The representative image of H-Cell during real time operation

Supplement: S2 Fig — (DOCX) [file pone.0332699.s002.docx]
